# Differential Contribution of NF-κB Signaling Pathways to CD4^+^ Memory T Cell Induced Activation of Endothelial Cells

**DOI:** 10.3389/fimmu.2022.860327

**Published:** 2022-06-13

**Authors:** Kim C. M. Jeucken, Charlotte C. N. van Rooijen, Yik Y. Kan, Lotte A. Kocken, Aldo Jongejan, Abraham C. I. van Steen, Jaap D. van Buul, Henric K. Olsson, Jan Piet van Hamburg, Sander W. Tas

**Affiliations:** ^1^ Department of Experimental Immunology, Amsterdam University Medical Centers, University of Amsterdam, Amsterdam, Netherlands; ^2^ Department of Clinical Immunology and Rheumatology, Amsterdam Rheumatology and Immunology Center, Amsterdam University Medical Centers, University of Amsterdam, Amsterdam, Netherlands; ^3^ Department of Epidemiology and Data Science, Bioinformatics Laboratory, Amsterdam Public Health Research Institute, Amsterdam UMC, University of Amsterdam, Amsterdam, Netherlands; ^4^ Molecular Cell Biology Lab at Dept. Molecular Hematology, Sanquin Research and Landsteiner Laboratory, Amsterdam, Netherlands; ^5^ Leeuwenhoek Centre for Advanced Microscopy (LCAM), Section Molecular Cytology, Swammerdam Institute for Life Sciences (SILS), University of Amsterdam, Amsterdam, Netherlands; ^6^ Translational Science and Experimental Medicine, Research and Early Development, Respiratory & Immunology, BioPharmaceuticals R&D, AstraZeneca, Gothenburg, Sweden

**Keywords:** endothelial (dys)function, inflammation, immune mediated disorders, CD4+ memory T cells, NF-kB signaling, cellular interaction, transendothelial migration

## Abstract

Endothelial cells (ECs) are important contributors to inflammation in immune-mediated inflammatory diseases (IMIDs). In this study, we examined whether CD4^+^ memory T (T_m_) cells can drive EC inflammatory responses. Human T_m_ cells produced ligands that induced inflammatory responses in human umbilical vein EC as exemplified by increased expression of inflammatory mediators including chemokines and adhesion molecules. NF-κB, a key regulator of EC activation, was induced by T_m_ cell ligands. We dissected the relative contribution of canonical and non-canonical NF-κB signaling to T_m_ induced EC responses using pharmacological small molecule inhibitors of IKKβ (iIKKβ) or NF-κB inducing kinase (iNIK). RNA sequencing revealed substantial overlap in IKKβ and NIK regulated genes (n=549) that were involved in inflammatory and immune responses, including cytokines (IL-1β, IL-6, GM-CSF) and chemokines (CXCL5, CXCL1). NIK regulated genes were more restricted, as 332 genes were uniquely affected by iNIK versus 749 genes by iIKKβ, the latter including genes involved in metabolism, proliferation and leukocyte adhesion (VCAM-1, ICAM-1). The functional importance of NIK and IKKβ in EC activation was confirmed by transendothelial migration assays with neutrophils, demonstrating stronger inhibitory effects of iIKKβ compared to iNIK. Importantly, iIKKβ – and to some extent iNIK - potentiated the effects of currently employed therapies for IMIDs, like JAK inhibitors and anti-IL-17 antibodies, on EC inflammatory responses. These data demonstrate that inhibition of NF-κB signaling results in modulation of T_m_ cell-induced EC responses and highlight the potential of small molecule NF-κB inhibitors as a novel treatment strategy to target EC inflammatory responses in IMIDs.

## Highlights

- NF-κB signaling critically involved in EC activation induced by CD4^+^ memory T cells
- Canonical and non-canonical NF-κB signaling have overlapping and different roles
- NF-κB signaling is a potential new target for treatment of EC inflammatory responses

## Introduction

Inflammation is an important hallmark of many immune mediated and autoimmune diseases like rheumatoid arthritis (RA) and vasculitis. Originally, research on immune-mediated inflammatory diseases (IMIDs) focused mainly on immune cells, but various stromal cells - including endothelial cells (ECs) - are increasingly recognized as essential players in inflammatory conditions and even as orchestrators of local immune responses (1–3). Under normal circumstances, ECs maintain vessel wall integrity and deliver oxygen and nutrients to tissues. However, once activated, ECs actively contribute to inflammation *via* various processes, such as angiogenesis, antigen presentation and immune cell recruitment (2, 3).

EC responses can be triggered by leukocytes, including CD4^+^ memory T (T_m_) cells. Aberrant T_m_ cell functions are implicated in pathogenesis of various IMIDs, including RA and vasculitis (4–9). In general, T_m_ cell populations show disturbances in both function and frequency of different subtypes; i.e. many IMIDs are characterized by a decrease in regulatory T cell number and/or function, leading to expansion and uncontrolled function of effector memory T cells that produce pro-inflammatory cytokines like TNFα, IFNγ and IL-17 (6, 10). Moreover, specific T_m_ cells are identified as main contributors to chronic inflammation in IMIDs; for example, T helper 17 cells are increased both in RA and vasculitis patients and participate in (local) inflammation by secreting pro-inflammatory cytokines, steering auto-antibody B cell responses and interacting with local stromal cells (5–7, 10). Given their central role in inflammation, is not surprising that many drugs currently used to treat IMIDs affect T cell interactions or cytokines produced by these cells, for example anti-TNFα and -IL-17 monoclonal antibodies (6, 10).

Since both T_m_ cells and ECs contribute to inflammation, characterizing the interactions between these cells is of particular relevance. Besides direct contact between EC and T_m_ cells, soluble mediators, like IL-6, TNFα, IL-17, produced by T_m_ cells are capable to trigger EC activation leading to expression of cytokines and adhesion molecules (11–15). Several studies showed that stimulation of ECs with individual pro-inflammatory stimuli, including those produced by activated T_m_ cells, (i.e. TNFα, lymphotoxin β (LTβ), IFNγ, IL-17), trigger signal transduction pathways that drive ECs inflammatory responses (14, 16–18). Among others, PI3K, JAK-STAT, MAPK and NF-κB signaling have been demonstrated to regulate the production of inflammatory mediators, pathological angiogenesis, antigen presentation and interactions with immune cells by ECs (2, 3). Thus, cytokines produced by T_m_ cells can trigger EC activation but – importantly - under pathological conditions T_m_ cells are not limited to the production of individual factors but rather produce a wide range of soluble factors. However, an extensive understanding of the effects of these complex combinations of soluble factors on EC activation is still lacking. In this study, we used whole transcriptome analysis to identify genes and key signaling pathways involved in EC activation by T_m_ cell produced soluble factors. We identified an important role for NF-κB signaling in these responses and further dissected the relative contributions of the canonical and non-canonical NF-κB pathways. Lastly, we explored the therapeutic potential and possible additive effects of NF-κB inhibition over currently used targeted therapies for IMIDs on EC inflammatory responses.

## Methods

### Subjects

Peripheral blood mononuclear cells (PBMCs) were isolated from buffy coats of healthy donors (Sanquin, Amsterdam) using gradient centrifugation on Lymphoprep (Frenesius Kabi). Subsequently, CD4^+^ T_m_ cells were isolated from PBMCs using CD4^+^ T_m_ cell magnetic-activated cell sorting (MACS) kit (Miltenyi) according to manufacturer’s instructions (purity > 90%). Human umbilical vein endothelial cells (HUVECs) were obtained from healthy donors (Amsterdam UMC, location AMC, Department of Obstetrics) or, for the TEM assay (as described below), purchased from Lonza. Neutrophils were isolated from whole-blood derived from healthy donors (Sanquin, Amsterdam) as described previously (19). Patient consent was obtained from all participants in written format according to the Declaration of Helsinki and approved by the medical ethics committee of the Academic Medical Centre, University of Amsterdam, Amsterdam, the Netherlands.

### Reagents

Pharmacological inhibitors of IKKβ (iIKKβ) and NIK (iNIK) were resynthesized by AstraZeneca (Gothenburg, Sweden) following patented procedures (20, 21). Detailed information on the inhibitors regarding structure and specificity can be found in **Supplementary Figure 1**, additional data on toxicity and specificity of iNIK was previously published in references (16) and (22). Unless otherwise stated, concentrations of 1.0 µM iIKKβ and 2.5 µM iNIK were chosen for optimal effects without evidence for toxicity, as described previously (16, 22). Inhibitor for JAK (tofacitinib, Sigma-Aldrich) was dissolved according to manufacturers’ instructions and used at a final concentration of 1.0 µM. TNFα blocker (etanercept (Pfizer)), anti-IL-17 (secukinumab (Novartis)) and anti-IL-6R (tocilizumab (Roche)), were dissolved in Medium 199 (M199; Gibco) to a concentration of 10 mg/ml and used at final concentrations of 10 µg/ml.

### Cell Culture

Purified T_m_ cells (1.0x10^5^) were cultured in RPMI 1640 containing 10% FCS, 0,1 μg/ml penicillin and streptomycin (pen/strep; Gibco), 10 mM L-glutamine (Lonza, Switzerland) and 50 μM β-mercaptoethanol (Merck, Darmstadt). Soluble anti-CD3 (purified from culture supernatant, 1:5000) and 5.0 μg/ml anti-CD28 (Sanquin) were added to cultures to activate T_m_ cells. After 72h, culture supernatant (T_m_ sup) was collected, filtered (syringe filter; 0.2 µm pore size, Sigma-Aldrich) to eliminate cell debris, pooled from 4-5 donors and stored at -20°C until further use.

HUVECs were cultured on 1% gelatin/PBS-coated culture plates in M199 containing 20% fetal calf serum (FCS; Biowest) 0.1 μg/ml penicillin and streptomycin (pen/strep; Gibco), 10 mM L-glutamine (Lonza), 0.025 U/ml heparin and 18.75 ng/ml EC growth serum (ECGS; Gibco). Cells were passaged using Trypsin/EDTA (0.05% in PBS) and used for assays up to passage 5. Prior to stimulation assays, HUVECs were seeded on 1% gelatin coated plates and left to adhere overnight. Next, cells were stimulated with T_m_ sup for 24-72h depending on the experiment. HUVECs were pre-incubated with inhibitors and DMARDs 2h prior to start of stimulation. In experiments where inhibitors of DMARDs were used, DMSO was used as control treatment.

### siRNA Transfection

HUVEC were transfected with non-targeting, IKKβ-targeting or NIK- targeting siRNA at 50 nM final concentration (GE Dharmacon, Pittsburgh, PA, USA) using Dharmafect I (GE Dharmacon, Pittsburgh, PA, USA) as previously described (22).

### Transendothelial Migration Assay

HUVECs (P1052, Lonza) were cultured in fibronectin-coated μ-slides VI0.4 (Ibidi) with 3x10^4^ cells per channel and stimulated overnight with T_m_ sup. Freshly isolated neutrophils were resuspended at 1.0×10^6^ cells/ml in HEPES medium (pH 7.4), stained with Vybrant DiD (Invitrogen) and activated for 20 min at 37°C and washed with HEPES. Ibidi flow chambers were connected to a perfusion system and exposed to 0.5 ml/min HEPES medium (pH 7.4) leading to a physiological shear flow of 0.8 dyn/cm^2^ for 5 min before injection of neutrophils into the perfusion system. Neutrophil-endothelial interactions were recorded for 20 min at 0.2 frames per second by a Zeiss Axiovert 200M microscope (Zeiss). Imaging was performed at 37°C in the presence of 5% CO2 at shear flow 0.8 dyn/cm^2^. The number of transmigrated neutrophils was quantified using ImageJ software (NIH).

### Cytokine Measurements

Cytokine antibody array (ab133998, Abcam) technology was used to analyze cytokines present in T_m_ sup. Proteins were visualized using ImageQuant LAS 4000 imaging system (GE Healthcare) and relative pixel density was measured using ImageJ software. ELISA was performed to determine CXCL5, IL-6, TNFα, LIGHT and LTβ concentrations in culture supernatants (CXCL5 and LIGHT; R&D systems, IL-6 and TNFα; U-CyTech, LTβ; MyBioSource). Cytokine concentrations were measured using microplate reader 680 (Biorad). A custom Magnetic Luminex assay (R&D) was designed to analyze concentrations of the following cytokines and chemokines in culture supernatants: CCL3, CCL8, CCL20, CXCL1, CXCL6, G-CSF, GM-CSF, IL-1β, TNFFSF18, VEGF, VEGF-C. Measurement of cytokine concentrations was done using Bio-plex 200 reader (Biorad). All cytokine measurements were performed according to manufacturer’s instructions.

### Protein Isolation and Western Blot Analysis

Cells were lysed in ice-cold radioimmunoprecipitation assay (RIPA) buffer supplemented with protease inhibitor (1:100; ThermoFisher Scientific). Protein concentrations were measured using Pierce™ BCA Protein Assay Kit (ThermoFisher Scientific) according to manufacturer’s instructions and equal amounts of protein were separated using SDS-PAGE and transferred to nitrocellulose membrane (ThermoFisher Scientific). Membranes were blocked with 2.5% Blotting-Grade Blocker (Biorad) in tris-buffered saline-Tween (TBST) and probed overnight with primary antibodies for p100/p52 (Mouse anti-NFKappaB p52, Millipore), p-IκBα (Mouse anti-Phospho-IkappaBalpha, Cell Signaling), VCAM-1 (mouse anti-CD106, Biolegend), ICAM-1 (mouse anti-CD54, BD) or E-selectin (rabbit anti-CD62E, Abcam) in 2.5% Blotting-Grade Blocker/TBST. Membranes were extensively washed and probed with HRP-conjugated secondary antibodies (goat anti-mouse or swine anti-rabbit, Agilent Dako) in 5% Blocking Grade buffer/TBST. Proteins were visualized using LUMI-LIGHT^PLUS^ (Roche) and ImageQuant LAS 4000 imaging system (GE Healthcare).

### RNA Isolation and Quantitative RT-PCR

RNA was isolated using GenElute™ Mammalian Total RNA Miniprep (Sigma-Aldrich; RT-PCR samples) or RNA Nucleospin XS kit (Bioké; RNA sequencing samples) according to manufacturer’s instructions. cDNA was synthesized using ThermoFisher Scientific first strand cDNA synthesis kit (ThermoFisher Scientific). Quantative RT-PCR was done by SybrGreen assay (Applied Biosystems) with an input of 5 ng cDNA and appropriate primers (primer sequences available upon request). Analysis was done using StepOne Plus (Applied Biosystems).

### RNA Sequencing and Data Analysis

RNA quality check was done using TapeStation RNA ScreenTape (Agilent). Samples with a minimal RNA integrity number of 9 (out of 10) were further processed for RNA sequencing. cDNA synthesis and library preparation were done according to Kapa mRNA Hyperprep protocol. Single-end 50 base pair sequencing (40x10^^6^ reads per sample) was performed using Illumina HiSeq4000 system at Amsterdam UMC. Reads were subjected to quality control (FastQC; v0.11.15, dupRadar; v1.0.0), trimmed for adapter sequences (Trimmomatic; v0.32) and aligned to the UCSC hg38 genome using HISAT2 (v2.1.0). Counts were obtained using HTSeq (v0.11.0) using the corresponding GTF (Ensembl; v94). Statistical analyses were performed using the edgeR and limma/voom R packages. Genes with more than 2 reads in at least 4 samples were kept. Count data were transformed to log2-counts per million (logCPM), normalized by the trimmed mean of M-values^31^ and precision weighted using voom (linear model framework; limma, R). Donor ID was used as a blocking factor to account for inter-donor treatment variability. Geneset enrichment was performed using CAMERA with a preset value of 0.01 for the inter-gene correlation using collections H, C1, C2, C3, C5, C6, and C7 retrieved from the Molecular Signatures Database (MSigDB v7.0; Entrez Gene ID version).

### Statistical Analysis

For RNA sequencing data, statistical analyses were performed using R (edgeR, limma/voom packages) for differential expression (DE) analysis (ANOVA model). For all other experiments, statistical significance was determined using Student’s two-tailed ratio paired t-test using GraphPad Prism (v8.2.1; Graphpad Software Inc.) for all assays unless differently stated. Differences were considered significant when *p* values <0.05. Significance is stated as follows: *p ≤ 0.05; **p ≤ 0.01; ***p ≤ 0.001; ****p ≤ 0.0001. Mean and SEM are shown, unless otherwise stated.

## Results

### Activated CD4^+^ Memory T Cells Produce Ligands That Effectively Induce Inflammatory Responses in Endothelial Cells

**Figure 1:**
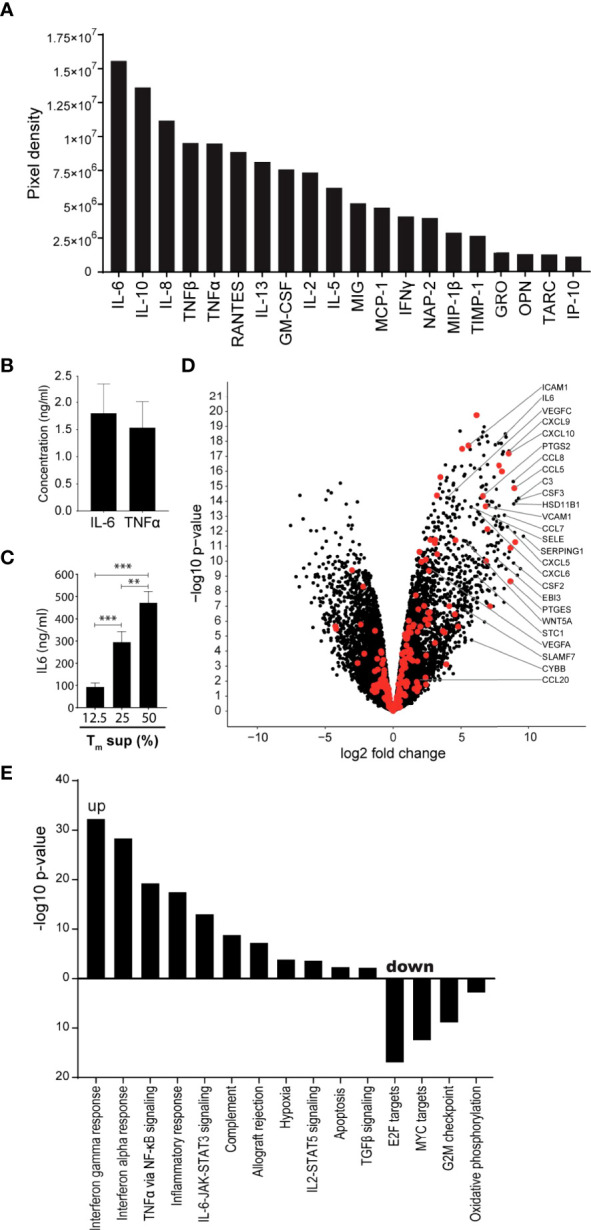
T_m_ sup stimulation drives inflammatory activation of ECs. **(A, B)** Purified Tm cells were cultured in presence of anti-CD3 and anti-CD28 for 72h after which culture supernatant was collected and pooled for 5 donors (T_m_ sup). **(A)** Relative abundance, rated by pixel density, of indicated cytokines in T_m_ sup measured by cytokine antibody array technology. **(B)** Quantification of IL-6 and TNFα concentrations in T_m_ sup by ELISA. Mean and SEM of experimental duplos is shown. **(C)** ECs were stimulated with T_m_ sup for 72h. Quantification of IL-6 protein production by ECs (*n=*6). Control samples (RPMI medium) or unstimulated ECs did not produce detectable levels of IL-6 (not shown). **(D, E)** RNA sequencing was performed on ECs after 72h of T_m_ sup stimulation. **(D)** Volcano plot showing changes in gene expression in T_m_ sup stimulated vs unstimulated ECs. Enrichment (x-axis, log2 fold change) is plotted against significance (y-axis, -log10 *p*-value). Selected genes were annotated and NF-κB associated genes are highlighted in red. **(E)** Differentially expressed Hallmark gene sets in T_m_ sup stimulated vs unstimulated ECs. For MYC targets, Hallmark gene set *MYC targets V2* is shown, similar results were obtained for *MYC targets V1*. -log10 *p*-value of 1.3 corresponds to *p=*0.05. **p < 0.01, ***p < 0.001.

T_m_ cells are known producers of cytokines, chemokines and growth factors that can induce EC activation (16, 23, 24). Using cytokine array technology, we confirmed that culture supernatant of anti-CD3/CD28 activated T_m_ cells (T_m_ sup) contain high levels of inflammatory cytokines capable of inducing EC activation, including IL-6, IL-8 and TNFα, as well as the anti-inflammatory cytokine IL-10 and several growth factors including CCL5/RANTES and GM-CSF (**Figure 1A**). Since IL-6 and TNFα are well-known to induce EC activation, concentrations of these proteins were quantified in T_m_ sup and found to be 1.80 ng/ml for IL-6 and 1.53 ng/ml for TNFα (**Figure 1B**).

The presence of a multitude of pro-inflammatory factors suggested that T_m_ sup stimulation had the potential to induce inflammatory responses in EC. To validate this, IL-6 production by EC stimulated for 72h with T_m_ sup was measured as an indicator for inflammatory activation. We found a concentration dependent increase of IL-6 production by T_m_ sup stimulated ECs (**Figure 1C**), which was >100-fold higher than the IL-6 concentration found in T_m_ sup. Since stimulation with 50% T_m_ sup induced strongest IL-6 production we continued with this concentration.

To dissect the effects of T_m_ sup stimulation on the transcriptome level we performed RNA sequencing (RNAseq) on T_m_ sup stimulated ECs. Overall, stimulation with T_m_ sup had strong effects on EC gene expression; transcriptome analysis revealed differential expression of 4954 genes compared to unstimulated ECs(Log2FC>1, *p ≤* 0.05; 2417 up, 2537 down). Notably, the top 50 differentially expressed genes largely consisted of genes encoding inflammatory mediators, including *C3*, *CCL7*, *CCL8, CXCL9, CXCL10, SERPING1* and *PTGES* (**Supplementary Table I**). Moreover, RNA sequencing data revealed strong upregulation of genes downstream of the NF-κB signaling, which is strongly associated with EC inflammatory responses. This included increased expression of genes encoding for cytokines and chemokines like *CSF2, CSF3, CCL5* and *CXCL5*, as well as adhesion molecules like *SELE, VCAM1* and *ICAM1* (**Figure 1D**).

Hallmark gene sets represent well-defined biological processes and responses making them an excellent tool to assess changes in cellular processes (25). Sixteen Hallmark gene sets were differentially expressed in EC after T_m_ sup stimulation (*p ≤* 0.05, false discovery rate (FDR) ≤0.05) (11 up, 5 down). Upregulated gene sets included those relating to immune reactions, most strongly interferon and inflammatory responses. Importantly, several signaling routes were prominently induced upon T_m_ sup stimulation, most notably *IL-6-JAK-STAT signaling* and *TNFα via NF-κB signaling*. Downregulated gene sets, i.e. MYC targets and oxidative phosphorylation, mainly associated with proliferation and metabolism (**Figure 1E**).

Together, these data indicate that stimulating ECs with T_m_ sup induces transcription of genes that are particularly involved inflammatory responses, including high upregulation of NF-κB (target) genes. Given the prominent role of NF-κB signaling in EC inflammatory responses, dissecting the contribution of both canonical and non-canonical NF-κB signaling to T_m_ sup-mediated EC activation is of particular interest.

### T_m_ sup Stimulation Induces Both Canonical and Non-Canonical NF-κB Signaling in Endothelial Cells

Previous studies have established a central role of NF-κB signaling in EC inflammatory reactions (16, 26–28). NF-κB signaling can follow two distinct signaling pathways referred to as the canonical and non-canonical pathways (reviewed extensively in (29)). In short, the canonical NF-κB pathway depends on activation of the IκB kinase (IKK) complex - consisting of IKKβ, IKKα, and IKKγ (or NEMO) – which induces phosphorylation of IκBα and downstream signaling. Non-canonical NF-κB signaling depends on accumulation of NF-κB inducing kinase (NIK) and downstream p100 to p52 processing (16, 29). We and others demonstrated that triggering of TNF receptor superfamily (TNFRSF) members, including LTβR, results in activation of both canonical and non-canonical NF-κB signaling in ECs (26, 30–32). Since RNA sequencing data revealed strong upregulation of NF-κB target genes in ECs in response to T_m_ sup stimulation, we analyzed putative induction of canonical and non-canonical NF-κB signaling in EC in more detail.

**Figure 2:**
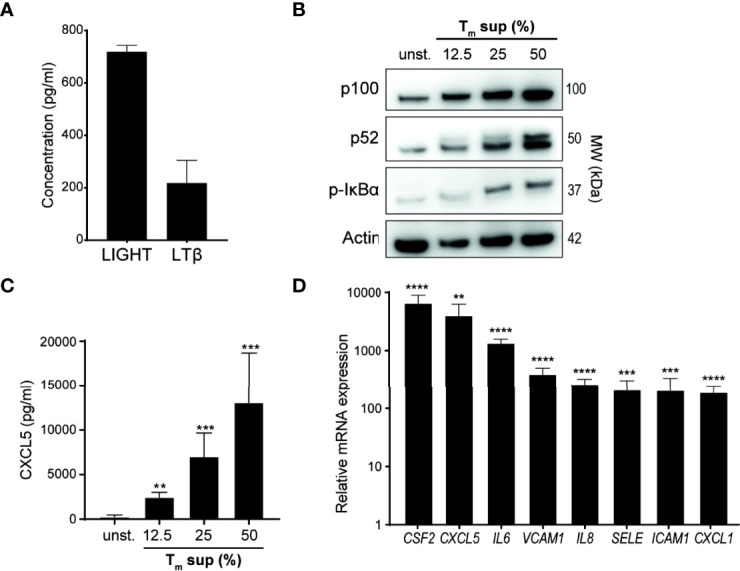
Tm sup stimulation activates NF-κB signaling in ECs. **(A)** Quantification of LTβ and LIGHT protein levels in pooled T_m_ sup (*n=*5) by ELISA. Mean and SEM of experimental triplos is shown. **(B-D)** EC were stimulated with T_m_ sup for 72h. **(B)** Representative western blot image of p100, p52 and p-IκBα expression in unstimulated (unst.) and stimulated ECs. Actin served as loading control. **(C)** ELISA quantification of CXCL5 protein production by ECs (*n=*6). Unstimulated ECs did not produce detectable levels of CXCL5 (not shown). **(D)** qPCR quantification of indicated mRNA transcripts in ECs stimulated with 50% T_m_ sup (*n=*5). **p < 0.01, ***p < 0.001, ****p < 0.0001.

In addition to measuring TNF levels (**Figure 1B**), we also quantified levels of the TNFSF members LTβ and LIGHT in T_m_ sup. LTβ and LIGHT concentrations were 218 pg/ml and 719 pg/ml, respectively (**Figure 2A**).

Next, we evaluated activation of canonical and non-canonical NF-κB signaling in T_m_ sup stimulated ECs. T_m_ sup stimulation induced dose-dependent activation of both canonical (increase in phosphorylated (p-)IκBα) and non-canonical (increased p100 to p52 processing) NF-κB signaling (**Figure 2B**). To confirm protein production of NF-κB target genes, we measured CXCL5 levels produced by T_m_ sup-stimulated EC. In line with activation of NF-κB signaling, a concentration dependent increase in CXCL5 production was observed (**Figure 2C**).

We previously showed that EC-NF-κB signaling in response to specific TNFRSF member stimulation leads to transcription of target genes, including various chemokines and cytokines (*CXCL1*, *CXCL5*, *IL-6*, *IL8* and *CSF2*) and adhesion molecules (*VCAM1*, *ICAM1, SELE*) *(* 16). Similarly, mRNA analysis demonstrated that expression of these genes by T_m_ sup stimulated ECs was up to 1.0x10^4^ times higher than in unstimulated ECs (**Figure 2D**).

Collectively, these data show that T_m_ cell produced ligands can drive both canonical and non-canonical NF-κB signaling and induce production of downstream inflammatory mediators in EC.

### Treatment With IKKβ and NIK Inhibitors Reduces T_m_ sup Mediated NF-κB Signaling

**Figure 3:**
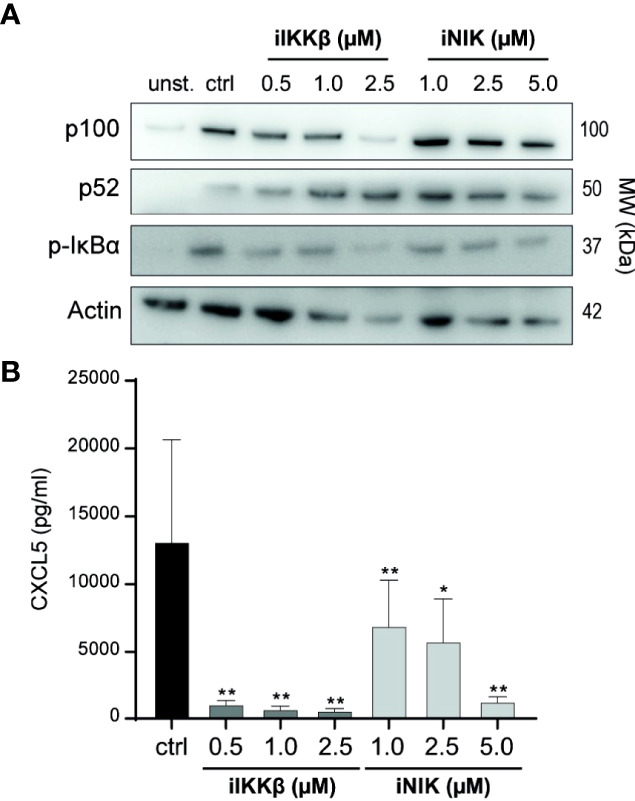
Treatment with small molecule inhibitors of IKKβ and NIK reduces NF-κB signaling in T_m_ sup stimulated ECs. **(A, B)** ECs were left unstimulated (unst.) or stimulated with T_m_ sup for 72h and treated with DMSO (ctrl) or with inhibitors of IKKβ (iIKKβ) or NIK (iNIK) at different concentrations. **(A)** Representative western blot image of p100, p52 and p-IκBα expression in ECs, actin served as loading control. **(B)** ELISA quantification of CXCL5 protein production by ECs (*n=*5). Dark gray; iIKKβ, light gray; iNIK. **p < 0.01, ***p < 0.001.

In earlier studies, we described the capacity of small molecule inhibitors of IKKβ (iIKKβ) and NIK (iNIK) to block canonical and non-canonical NF-κB signaling respectively (16, 22). To confirm this potential in T_m_ sup stimulated ECs, we probed for NF-κB signaling after treatment with iIKKβ and iNIK. Blocking of IKKβ resulted in a dose-dependent reduction of canonical NF-κB signaling (decrease in p-IκBα) without affecting non-canonical NF-κB signaling. Inhibition of NIK resulted in a dose-dependent reduction of both non-canonical (decrease in p100 to p52 processing), and canonical NF-κB signaling (decrease in p-IκBα), which is in line with previous results demonstrating a role for NIK in both non-canonical and canonical NF-κB signaling (**Figure 3A**) (16). To verify these results on protein production of NF-κB target genes, CXCL5 production by ECs treated with NF-κB inhibitors was analyzed. Both iIKKβ and iNIK reduced CXCL5 production in a concentration dependent manner in T_m_ sup stimulated ECs (**Figure 3B**). Together, these findings show that both IKKβ and NIK are critically involved in regulating T_m_ sup-induced EC responses.

### Overlapping and Distinct Contributions of NIK and IKKβ to T_m_ sup-Mediated Endothelial Cell Activation

**Figure 4:**
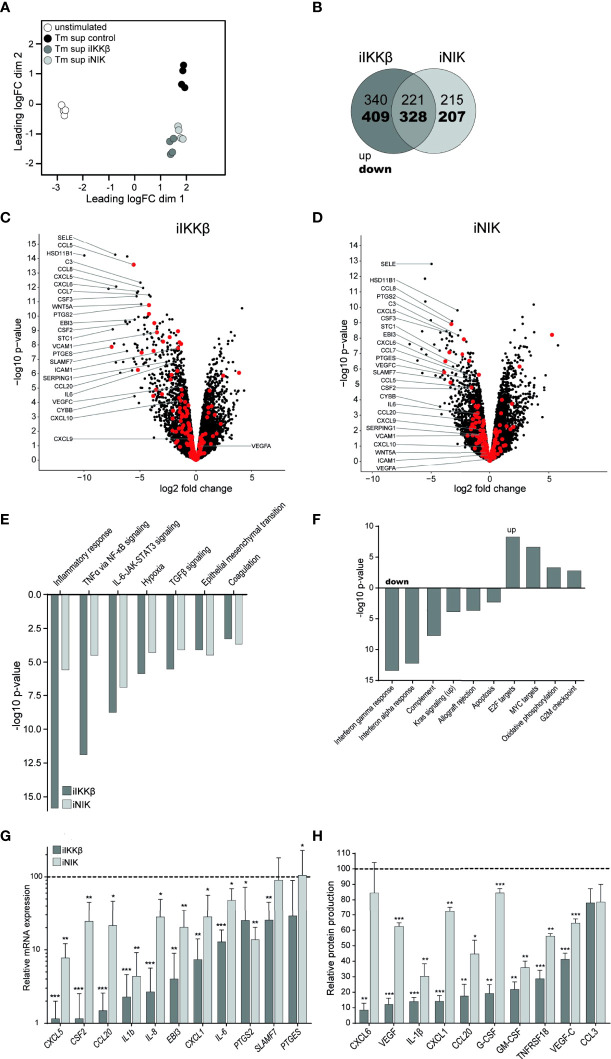
Treatment with NF-κB inhibitors reduces EC inflammatory activation. RNA sequencing was performed on ECs after 72h of T_m_ stimulation and treatment with iIKKβ or iNIK. **(A)** Clustering of different RNA sequencing conditions. Multi-dimensional scaling of transcriptomes based on top 500 genes in unstimulated (white), T_m_ stimulated DMSO treated (control, black), T_m_ stimulated 1.0 µM iIKKβ treated (dark gray) and T_m_ stimulated 2.5 µM iNIK treated (light gray) ECs (*n=*4). Each dot represents an individual sample. **(B)** Number of genes differentially expressed in T_m_ sup stimulated ECs treated with iIKKβ (dark gray), iNIK (light gray) or both (pink) compared to control (log2 fold change>1.0, *p*<0.05). **(C, D)** Volcano plot showing changes in gene expression in **(C)** T_m_ stimulated iIKKβ treated vs T_m_ stimulated DMSO treated (control) ECs and **(D)** T_m_ stimulated iNIK treated vs control ECs. Enrichment (x-axis, log2 fold change) is plotted against significance (y-axis, -log10 *p*-value). Selected genes are annotated and NF-κB associated genes are highlighted in red. **(E, F)** Analysis of Hallmark gene sets. **(E)** Differentially expressed (FDR ≤ 0.05, p<0.05) Hallmark gene sets in both T_m_ stimulated iIKKβ treated and iNIK treated ECs vs control ECs. **(F)** Hallmark gene sets exclusively affected in T_m_ sup stimulated iIKKβ treated ECs vs control ECs (FDR ≤ 0.05, p<0.05). For MYC targets, Hallmark gene set *MYC targets V2* is shown, similar results were obtained for *MYC targets V1*. RNA sequencing results were validated on a selection of pro-inflammatory cytokines, chemokines, growth factors and other inflammatory mediators on **(G)** mRNA level by qPCR and **(H)** protein level by Magnetic Luminex assay (*n=*5). Dark gray; iIKKβ, light gray; iNIK. Values are shown relative to control (dashed line). *p < 0.05, **p < 0.01, ***p < 0.001.

To dissect the roles of IKKβ and NIK in T_m_ sup mediated EC responses we used RNAseq to analyze changes in gene (set) expression in ECs treated with iIKKβ and iNIK. Global differences in gene expression of iIKKβ and iNIK treated ECs were investigated using multi-dimensional scaling analysis. This revealed clear differences in transcriptome profiles of all tested culture conditions (**Figure 4A**).

Treatment with iIKKβ led to differential expression of 1298 genes (Log2FC>1, *p ≤* 0.05; 561 up, 737 down). In comparison, treatment with iNIK affected the transcription of ~25% less genes, namely 971 (436 up, 535 down). Notably, there was a substantial overlap of genes regulated by both IKKβ and NIK (549; 221 up, 328 down), indicating transcriptional similarities downstream of these kinases (**Figure 4B**). Among these genes, in particular transcripts for inflammatory mediators were present, including chemokines (i.e. *CSF3*, *CXCL5*, *CCL8*, *PTGES*) and adhesion molecules (i.e. *SELE*) (**Figures 4C, D**; **Supplementary Table I**). Interestingly, although expression of some adhesion molecules (i.e. *SELE*) was dependent on both IKKβ and NIK signaling, expression of others (i.e. *VCAM1, ICAM1*) depended solely on IKKβ. Thus, IKKβ appears to have a broader effect on gene expression compared to NIK.

To gain insight into the cellular processes regulated by IKKβ and NIK, we performed Hallmark gene set enrichment analysis. IKKβ inhibition resulted in differential expression of 18 gene sets (5 up, 13 down), whereas NIK inhibition resulted in differential expression of 7 gene sets (all down) (*p ≤* 0.05, FDR ≤0.05). Interestingly, no Hallmark gene sets were exclusively affected by NIK inhibition. Gene sets regulated *via* both IKKβ and NIK predominantly related to inflammatory responses and signaling pathways, most importantly *TNFα via NF-κB signaling* and *IL-6-JAK-STAT3 signaling* (**Figure 4E**). Gene sets exclusively regulated by IKKβ included additional immune responses such as interferon alpha and gamma response and complement pathway (down),cellular proliferation (i.e. MYC targets) and oxidative phosphorylation (up) (**Figure 4F**).

Next, we validated the results obtained by whole transcriptome analysis by measuring mRNA expression and protein production of a selection of cytokines (i.e. IL-6, IL-1β), chemokines (i.e. CXCL1, CXCL5), growth factors (i.e. G-CSF, GM-CSF) and other genes associated with inflammation (PTGES, PTGS) (**Figures 4G, H**). Similar to the RNAseq-derived data, we found that both IKKβ and NIK regulated expression of these inflammatory mediators, and that interfering with IKKβ reduced expression most strongly. To confirm specificity of used inhibitors, results were validated by stimulating HUVEC treated with siRNA targeting NIK or IKBK. Treatment with siRNA or inhibitors for NF-κB led to similar effects on expression of inflammatory mediators, confirming specificity of used inhibitors (**Supplementary Figure 2**).

Together, data derived from RNAseq and validation assays suggest a substantial overlap in genes and cellular processes depending on both IKKβ and NIK activation, mainly those relating to immune and inflammatory responses. Notably, the role of NIK seems to be restricted to inflammatory responses, whereas IKKβ is involved in regulating the expression of many additional genes and processes, including those associated with proliferation and metabolism.

### Additional Effect of iIKKβ Over Current IMIDs Therapies in Suppressing EC Inflammatory Activation

Collectively, the effects of iIKKβ and iNIK on EC inflammatory responses suggest that these inhibitors may have therapeutic potential in IMIDs. Therefore, we tested whether combination of these inhibitors with current targeted therapeutics for IMIDs may have additive value over single drug treatment.

**Figure 5:**
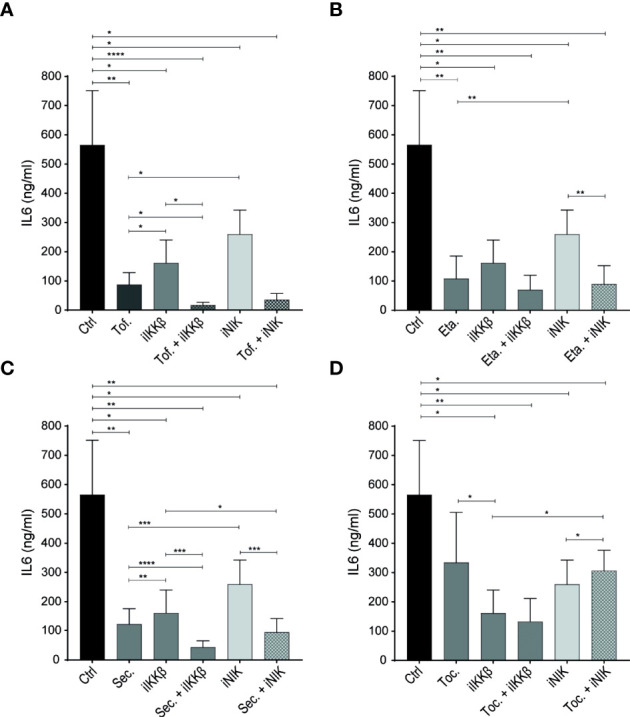
NF-κB inhibitors have additive effects over JAK inhibitors and biological DMARDs. **(A–D)** IL-6 production by ECs stimulated with 72h T_m_ sup and **(A)** treated with DMSO (control; ctrl), JAK inhibitor (tofacitinib; Tof.), iIKKβ, iNIK or a combination, or treated with **(B)** TNF blocker (etanercept; Eta.), **(C)** anti-IL-17 (secukinumab; Sec.) or **(D)** anti-IL-6R (tocilizumab; Toc.) as single treatment or in combination with iIKKβ or iNIK (*n=*5). In **(B–D)**. *p < 0.05, **p < 0.01, ***p < 0.001, ****p < 0.0001.

Treating ECs with the JAK inhibitor tofacitinib alone significantly reduced IL-6 production, confirming an important role for JAK-STAT signaling in EC inflammatory responses after T_m_ sup stimulation. Importantly, combining tofacitinib with iIKKβ or - to a lesser, non-significant extent – iNIK, potentiated the effects of either therapy alone (**Figure 5A**).

Next, we tested whether biologic DMARDs commonly used in treatment of IMIDs also had the potential to reduce EC inflammatory activation. Blocking TNF (by etanercept) and IL-17 (by secukinumab) significantly reduced IL-6 production (**Figures 5B, C**), confirming the potential of TNFα and IL-17 to trigger EC inflammatory activation (14, 15, 23). Moreover, combining anti-IL-17 with iIKKβ had additive effects over anti-IL-17 alone (**Figure 5D**).

Interestingly, we observed a limited effect of blocking IL-6R (by tocilizumab) in our system. Despite the high levels of IL-6 detected in T_m_ sup and the finding that IL-6-JAK-STAT3 signaling was strongly upregulated in response to T_m_ sup stimulation (**Figure 5D**).

### IKKβ Is the Main Regulator of Leukocyte Adhesion and Migration

**Figure 6:**
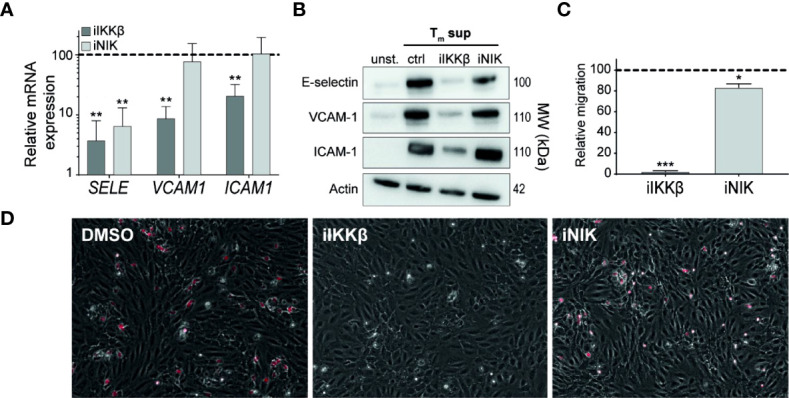
IKKβ is the main regulator of neutrophil adhesion and transendothelial migration. **(A, B)** ECs were left unstimulated or stimulated with T_m_ sup for 72h and treated with iIKKβ,iNIK or DMSO (control). **(A)** Quantification of indicated mRNA transcripts by qPCR (*n*=5). **(B)** Representative western blot image of indicated proteins. **(C, D)** ECs were stimulated for 24h with T_m_ sup and treated with iIKKβ, iNIK or DMSO (control). Next, a transmigration assay was performed with neutrophils. **(C)** Quantification of neutrophil migration and **(D)** representative images for indicated conditions; neutrophils attached to ECs are depicted in red. Dark gray; iIKKβ, light gray; iNIK. Values are shown relative to control (dashed line). *p < 0.05, **p < 0.01, ***p < 0.001.

Intravascular rolling, adhesion and transendothelial migration (TEM) of leukocytes depends strongly on adhesion molecules expressed on the surface of ECs. RNAseq-derived data on adhesion molecule expression revealed differential regulation of distinct adhesion molecules by IKKβ and NIK. In line with the RNAseq-derived data, validation of mRNA and protein expression showed that E-selectin was regulated both by IKKβ and NIK. In contrast, NIK appeared to be largely dispensable for expression of VCAM-1 and ICAM-1 expression (**Figures 6A, B**).

To analyze the effects of iIKKβ and iNIK on TEM of leukocytes, a TEM assay using neutrophils under flow conditions was performed. Neutrophil migration was almost completely blocked (97% reduction) after treatment with iIKKβ, whereas iNIK treatment reduced neutrophil migration to a lesser, but still significant, extent (17% reduction) (**Figures 6C, D**). Together, this indicates that leukocyte adherence to the endothelium and subsequent TEM is mainly dependent on canonical NF-κB signaling *via* IKKβ, but that there is still a role for NIK.

## Discussion

In this study we unraveled the mechanisms underlying EC responses driven by T_m_ cell soluble factors. We have identified a central role for NF-κB signaling in these responses and highlight the therapeutic potential of this pathway in treating IMIDs.

T_m_ cells have long been recognized for their role in IMIDs, including RA and vasculitis (6, 10). Moreover, identification of aberrant T_m_ cell responses led to the development of biologics that specifically target soluble factors produced by these cells, including anti-TNF, anti-IL-17 and anti-IL-6R monoclonal antibodies (6, 10). In contrast, EC activation and responses that contribute to inflammation have only started to be identified. T_m_ cells and their secreted soluble factors are capable of inducing EC activation, although it was hitherto not fully understood which mechanisms underlie T_m_ driven EC activation (11–15).

Here, we applied unbiased whole genome sequencing to identify key pathways and molecules in involved in EC activation driven by T_m_ cell soluble factors. We found that T_m_ sup stimulation of ECs led to a general increase in genes associated with inflammation and identified a prominent role for NF-κB and JAK-STAT signaling herein.

Since both canonical and non-canonical NF-κB signaling are widely recognized as central regulators of EC activation, we further explored the role of these pathways in T_m_ sup driven EC responses. By stimulating ECs with T_m_ sup in the presence of small molecule NF-κB inhibitors we identified both distinct and overlapping roles for IKKβ and NIK in regulating EC gene transcription. First, we found a substantial overlap between genes regulated *via* IKKβ and NIK, in particular those encoding for inflammatory mediators. In addition, we found genes that were exclusively regulated *via* IKKβ, including those associated with additional immune responses, apoptosis, proliferation and oxidative phosphorylation. Validation assays confirmed the results found by transcriptome analysis, and showed that expression of pro-inflammatory cytokines and chemokines is regulated both *via* IKKβ and NIK.

In contrast, expression of adhesion molecules appeared largely regulated by IKKβ, with only a limited role for NIK. These results were reflected in TEM functional assays showing a prominent role for IKKβ, and to a lesser extent for NIK, in the regulation of neutrophil adhesion and migration. These findings may seem to contradict earlier results based on experiment with LTβ and LIGHT stimulation of ECs in which NIK inhibition was as effective in blocking expression of adhesion molecules as IKKβ inhibition (16). However, this might be attributed to the high concentration of TNFα in T_m_ sup, as expression of adhesion molecules in response to TNFα is shown to be independent of NIK (33, 34). Since the TNFα concentration in T_m_ sup was up to 8 fold higher than those of LTβ and LIGHT, this may very well account for the prominent role of IKKβ found in our system.

Previously, we demonstrated that EC inflammatory responses depend on prolonged activation of canonical NF-κB signaling, regulated through the interaction of NIK with canonical NF-κB signaling (16). Given the substantial overlap in genes regulated *via* both IKKβ and NIK, we propose a similar mechanism here. Nonetheless, in this study IKKβ inhibition had overall stronger effects on individual gene expression than NIK inhibition. Additionally, a larger number of genes was affected by IKKβ inhibition in comparison to NIK inhibition. This broader effect may be partly explained by differences in the ligands activating the distinct NF-κB signaling routes; although all ligands activating NIK dependent NF-κB signaling can also induce canonical signaling, this is not the case vice versa (16, 35). The abundance of ligands specific for canonical NF-κB in T_m_ sup (i.e. TNFα and IFNγ) might contribute to the relative stronger effects of IKKβ inhibition found here. In addition, NIK dependent NF-κB signaling is slower compared to canonical NF-κB signaling (16). As a consequence, the effects of NIK inhibition only occur several hours after initial EC activation when the first wave of inflammatory mediators is already produced, whereas IKKβ inhibition has immediate effects and as such can also prevent initial cytokine production.

Classical NF-κB is one of the drivers of *P100* expression (32). Pure signal-induced (canonical-independent) p100 processing would present as an increase of p52 levels as proportional to a decrease of p100 levels, which was only demonstrated to a modest effect in our studies (**Figure 2**); we observed a concomitant increase in p100 and p52 levels, indicating increase in p52 may occur through normal basal processing of elevated p100 levels *via* canonical NF-κB signaling. This implies that non-canonical NF-κB signaling induced by T_m_ sup may be at least partly driven by canonical NF-κB signaling. Nevertheless, non-canonical NF-κB target genes are increased after T_m_ sup stimulation. In addition, the decrease in inflammatory mediators caused by iNIK indicates that effects of T_m_ sup stimulation also occur upstream of p100. Whether this is *via* NIK-mediated canonical or non-canonical NF-κB signaling remains to be fully elucidated, but our data clearly indicate that T_m_ sup induces EC activation *via* both canonical and NIK-mediated (or non-canonical) NF-κB signaling.

In addition to NF-κB signaling, JAK-STAT signaling was prominently upregulated in T_m_ sup stimulated EC. Since JAK-STAT signaling is an important therapeutic target in IMIDs and a known regulator of EC dysfunction, we further explored its role in T_m_ sup driven EC responses (2, 3, 36). We found that treating ECs with a JAK inhibitor strongly reduced IL-6 production and that combining JAK and NF-κB inhibitors potentiated the effects of either therapy alone. These additive effects are likely due to the existing overlap between NF-κB and JAK-STAT target genes, such as *IL8* and *CSF2* (15, 16). Importantly, RNAseq data revealed that interfering with NF-κB signaling also reduced JAK-STAT signaling, suggesting crosstalk between the two pathways. This crosstalk may occur at different stages of the signaling cascade. NF-κB signaling leads to the production of ligands for JAK-STAT signaling, for example IL-6 (37). Consequently, NF-κB inhibition will reduce availability of JAK-STAT ligands and as such hamper its signaling. An alternative, and more complicated, possibility is direct crosstalk between the NF-κB and JAK-STAT signaling routes, which may occur *via* various mechanisms, as is demonstrated in different cell types. For example; in esophageal squamous cell carcinoma abrogation of JAK-STAT3 signaling downregulates NF-κB signaling, in head and neck squamous cell carcinoma interfering with NF-κB reduced STAT3 signaling and in brain pericytes STAT3 cooperates with NF-κB to induce IL-6 production (38–40). This illustrates the many forms and complexity that crosstalk between pathways entails and further studies are needed to identify which mechanisms are at play in EC JAK-STAT- NF-κB crosstalk.

The T_m_ sup we used in this study contained a plethora of pro-inflammatory stimuli (i.e. TNFα, IL-6, LTβ, LIGHT). Blocking TNF and IL-17, but not IL-6, significantly reduced EC IL-6 production suggesting an important role for TNF and IL-17 in T_m_ cell-induced EC responses. In our system, combining anti-TNF and iIKKβ or iNIK did not have additive effects over anti-TNF alone, suggesting that TNF is the main driver of T_m_ sup mediated NF-κB signaling. We observed that blocking IL-17 reduced IL-6 production which to some extent contradicts an earlier study demonstrating that HUVECs are largely unresponsive to IL-17 stimulation (38). This discrepancy is might be due to differences in stimulation time; Liu et al. performed experiments by stimulating cells 6-12h, whereas we stimulated HUVECs for 72h. It is possible that IL-17 mediated effects only arise after longer stimulation times. Moreover, our T_m_ sup contained high concentrations of TNF and the combination of IL-17 and TNF stimulation has been reported to have synergistic effects in various cell types, including ECs (39, 40). Moreover, we found that combining anti-IL-17 and iIKKβ had additive effects over anti-IL-17 alone, indicating that IL-17 mediates EC responses *via* different or additional routes compared to those regulated *via* IKKβ. This may seem somewhat surprising, since IL-17 predominantly signals *via* the IL-17R-ACT-1/TRAF6 axis that induces NF-κB signaling (41). However, activation of JAK-STAT signaling in ECs in response to IL-17 has been shown (15). Given the strong activation of JAK-STAT pathway observed in our data set, IL-17 may activate JAK-STAT signaling in addition to NF-κB signaling. Surprisingly, although IL-6 was the highest expressed cytokine in Tm sup, blocking IL-6R had only minimal effects on EC activation. This might be explained by the fact that cells that express low levels of surface bound IL-6R, including ECs, can still activate the pathway. This activation depends on the presence of soluble (s)IL-6R to drive IL-6 trans-signaling, which is mediated *via* binding of IsIL-6R to IL-6/glycoprotein 130 complexes on the cell surface (42–44). It is possible that there are insufficient levels of sIL-6R present in Tm sup to drive robust IL-6 pathway activation and therefore the effects obtained by blocking IL-6R are only limited.

Although we did not explore the roles of IFNα and –γ, our data indicates that these cytokines contribute to EC activation since both IFN response pathways were strongly upregulated by T_m_ sup stimulation. In addition, other studies have shown that IFNγ can induce EC responses including complement activation, apoptosis, proliferation and oxidative phosphorylation (18, 41–44). Interestingly, all these responses were reversed when we treated ECs with iIKKβ, suggesting an important role for IFNγ in driving EC activation *via* NF-κB signaling.

Here, we focused on investigating the underlying mechanisms of EC responses driven by T_m_ cell soluble factors, and did not explore the contribution of direct cell-cell contact. However, in our experimental setting direct cellular contact between T_m_ cells and ECs did not intensify IL-6 or CXCL5 production compared to T_m_ sup stimulation (data not shown). Nonetheless, others have shown that direct interactions between EC and T cells induces expression of inflammatory mediators by ECs, indicating that although direct cellular contact is relevant, it is not a requirement for EC activation (11–13).

As our main interest was to identify the contribution of NF-κB signaling to EC inflammatory reactions in the context of chronic inflammation, we performed RNAseq on ECs stimulated for 72h. Previous studies indicated that after ~24-48h of stimulation both NF-κB pathways are important for EC activation, whereas NIK-mediated NF-κB is dispensable at earlier time points after stimulation (**Supplementary Figure 3**) (16, 22, 26). However, analysis of gene expression after 72h is complex since many secondary pathways will be activated, making it challenging to identify whether effects are mediated directly *via* NF-κB signaling or are (partly) explained by (autocrine or paracrine) stimulation of other signaling pathways. Adding to the complexity is that different inflammatory mediators follow different expression patterns. For example, chemokine expression increases steadily over time, while some adhesion molecules (i.e. *SELE*) have negative feedback loops with peak expressions earlier than studied here (**Supplementary Figure 3**). Nevertheless, we clearly show that both canonical and NIK-mediated NF-κB signaling are critically involved in (chronic) EC activation.

As we still face difficulties in treating IMIDs there is a need for additional treatment strategies to supplement existing therapies. In this respect, targeting IKKβ may be promising because it completely abrogated inflammatory responses and reversed other processes affected by T_m_ sup stimulation. Although more effective in our culture systems, targeting IKKβ might have potential harmful side-effects since canonical NF-κB signaling is also involved in many essential cellular processes (45, 46). Consequently, administering iIKKβ as a therapy for IMIDs could have unpredictable side-effects. In contrast, NIK-dependent signaling is more restricted to pathological processes, and as the effects of targeting NIK were limited to inflammatory responses without affecting essential processes, this is possibly a safer therapeutic strategy (47). Although more challenging, specific targeting of ECs is of particular interest as it would circumvent unwanted side-effects on other cell types while still reducing inflammation by impeding secretion of inflammatory mediators and recruitment of leukocytes by ECs. In addition, our preliminary data suggest that targeting of IKKβ or NIK might have additive effects over drugs currently used to treat IMIDs, including anti-TNF and anti-IL-17. Together, this highlights that NF-κB signaling is a main regulator of EC inflammatory responses and has potential as therapeutic target in patients with IMIDs, either as stand-alone treatment or in combination with existing therapies.

## Data Availability Statement

The data presented in the study are deposited in the GEO repository, accession number GSE203551.

## Author Contributions

Conceived the experiments: KJ, JH, and ST. Performed the experiments: KJ, LK, CR, YK, and AS. Analyzed the data: KJ, AJ, and AS. Wrote the original draft: KJ. Writing, review & editing: KJ, AJ, AS, JB, HO, JH, and ST. All authors contributed to the article and approved the submitted version.

## Funding

This work was supported by a Dutch Arthritis Society grant to ST (RF16-1-302) and by LSBR (#1649) and ZonMW NWO Vici grants (#91819632) to JB.

## Conflict of Interest

Author HO was employed by the company AstraZeneca.

The remaining authors declare that the research was conducted in the absence of any commercial or financial relationships that could be construed as a potential conflict of interest.

## Publisher’s Note

All claims expressed in this article are solely those of the authors and do not necessarily represent those of their affiliated organizations, or those of the publisher, the editors and the reviewers. Any product that may be evaluated in this article, or claim that may be made by its manufacturer, is not guaranteed or endorsed by the publisher.
