# Supplementary material for: Differential Contribution of NF-κB Signaling Pathways to CD4+ Memory T Cell Induced Activation of Endothelial Cells
Source: Front Immunol. 2022 Jun 13;13:860327. doi: 10.3389/fimmu.2022.860327 (PMC9235360; doi:10.3389/fimmu.2022.860327)
Supplement: Supplementary file 1 [file DataSheet_1.pdf]

Supplementary table I: Top 50 differentially expressed genes in T_m sup stimulated EC vs unstimulated EC and T_m sup stimulated EC treated with iIKKβ or iNIK vs T_m sup stimulated DMSO treated EC.

DMSO stimulated vs unstimulated[1]				iIKKβ vs DMSO[1]				iNIK vs DMSO[1]			
gene	LogFC	Pvalue	adjusted Pvalue	gene	LogFC	Pvalue	adjusted Pvalue	gene	LogFC	Pvalue	adjusted Pvalue
SERPING1	9.62	2.1E-12	2.3E-10	HSD11B1	-9.96	6.1E-15	4.9E-11	CCL3	-6.83	2.5E-08	1.7E-05
NEURL3	9.4	3.1E-10	1.6E-08	SERPINB7	-7.81	1.2E-12	3.2E-09	DCLK3	-6.17	2.8E-07	8.2E-05
C3	9.27	6.2E-15	1.7E-12	PTGES	-7.53	1.4E-08	3.3E-06	MRGPRX3	-5.83	5.2E-08	2.5E-05
CD7	9.18	8.7E-10	3.7E-08	STC1	-7.43	8.2E-10	4.3E-07	KLHDC7B	5.78	3.0E-08	1.8E-05
CSF3	9.1	9.2E-15	2.4E-12	CCL3	-7.30	7.8E-09	2.2E-06	IL1B	-5.77	3.6E-09	5.3E-06
MSC	9.02	5.3E-12	5.1E-10	SELE	-7.21	5.3E-15	4.9E-11	CCL3L1//CCL3L3	-5.76	1.8E-06	3.0E-04
C15orf48	9.01	1.2E-15	4.3E-13	CCL3L1//CCL3L3	-7.15	1.6E-07	2.2E-05	TNFRSF11B	-5.53	1.4E-12	1.4E-08
NA	8.97	1.2E-12	1.4E-10	CXCL5	-7.13	8.8E-13	3.0E-09	SERPINB7	-5.50	4.2E-11	2.9E-07
CCL5	8.96	1.3E-15	4.7E-13	CHI3L1	-6.76	8.7E-07	8.1E-05	XIRP1	-5.43	1.2E-07	4.4E-05
HSD11B1	8.92	1.5E-14	3.4E-12	DCLK3	-6.50	1.5E-07	2.1E-05	TNFSF18	5.27	6.1E-09	6.8E-06
CSF2	8.9	1.3E-10	7.4E-09	MRGPRX3	-6.44	9.4E-09	2.5E-06	PPFIA4	-5.04	7.0E-10	2.0E-06
CCL8	8.88	4.6E-16	2.2E-13	PPFIA4	-6.30	2.1E-10	1.6E-07	SELE	-4.98	1.5E-13	3.2E-09
SLAMF8	8.68	1.3E-11	1.1E-09	PRRX1	-6.28	2.5E-10	1.7E-07	CHI3L2	-4.75	2.6E-08	1.7E-05
PTGES	8.67	2.2E-09	7.9E-08	SLAMF7	-6.17	2.7E-08	5.2E-06	BEX2	4.73	2.6E-06	3.7E-04
OR2I1P	8.6	2.3E-11	1.8E-09	C15orf48	-6.14	7.2E-15	4.9E-11	CHRNA1	-4.64	2.6E-06	3.7E-04
CXCL9	8.57	4.2E-18	4.8E-15	CSF2	-6.13	3.4E-10	2.1E-07	NLRC4	4.62	2.5E-05	1.7E-03
CXCL10	8.55	6.2E-18	6.4E-15	AMPD3	-5.92	2.5E-10	1.7E-07	S100P	4.61	1.6E-05	1.2E-03
MSC-AS1	8.53	5.8E-11	3.9E-09	CCL20	-5.70	5.9E-07	6.1E-05	PROM1	4.46	1.5E-03	2.4E-02
CXCL5	8.46	4.9E-13	6.8E-11	XIRP1	-5.57	9.0E-08	1.4E-05	STC1	-4.29	6.4E-08	3.0E-05
GBP5	8.35	5.0E-19	1.3E-15	CCL5	-5.56	2.7E-14	1.4E-10	CYBB	-4.27	2.7E-04	7.9E-03
MX2	8.35	3.3E-10	1.6E-08	CYBB	-5.49	3.7E-05	1.3E-03	CPXM1	-4.24	3.4E-06	4.4E-04
C1R	8.31	3.1E-19	1.3E-15	SLC8A3	-5.47	1.5E-07	2.0E-05	PRRX1	-4.23	4.3E-09	5.6E-06
CCL7	8.24	8.1E-14	1.5E-11	EBI3	-5.47	1.7E-10	1.4E-07	OLR1	-4.13	8.5E-04	1.7E-02
AIM2	8.24	3.4E-11	2.5E-09	MCHR1	-5.46	8.3E-09	2.3E-06	P2RY6	-4.09	1.2E-08	9.9E-06
ACOD1	8.21	5.4E-08	1.2E-06	SERPINB4	-5.41	8.6E-09	2.3E-06	ALOX5AP	4.07	2.2E-04	6.8E-03
MIR3945HG	8.21	6.6E-09	2.0E-07	RUNX3	-5.34	2.2E-07	2.7E-05	NA	3.98	6.5E-05	3.3E-03
IDO1	8.16	4.6E-18	5.0E-15	CSF3	-5.31	4.1E-12	6.3E-09	PI3	-3.96	1.2E-06	2.4E-04
NA	8.11	5.5E-13	7.4E-11	CXCL8	-5.26	2.4E-12	4.4E-09	PTGES	-3.91	1.7E-06	2.9E-04
CXCL11	8.1	6.9E-18	6.8E-15	IL1B	-5.25	1.5E-08	3.6E-06	NA	-3.88	5.0E-06	5.8E-04
CD74	8.06	9.9E-17	6.3E-14	RN7SL124P	-5.23	1.1E-08	2.6E-06	NA	-3.87	1.6E-04	5.7E-03
SERPINB7	8.03	7.3E-13	9.2E-11	TNFRSF11B	-5.19	1.7E-12	3.9E-09	SLAMF7	-3.84	4.5E-06	5.4E-04
NOD2	8.02	1.9E-10	1.0E-08	EHF	-5.19	5.4E-07	5.8E-05	SP6	-3.80	3.2E-07	8.8E-05
TNFAIP6	8.01	4.0E-12	4.1E-10	CXCL6	-5.18	1.9E-12	3.9E-09	MAPK2K6	3.79	3.9E-06	4.9E-04
C1S	8	1.3E-18	2.2E-15	LINC01539	-5.16	2.5E-08	5.1E-06	NUPR1	3.76	4.2E-09	5.6E-06
CX3CL1	7.95	2.9E-13	4.3E-11	CSPG5	-5.15	9.3E-10	4.7E-07	OR6D1P	3.76	1.1E-05	9.9E-04
HLA-DRA	7.93	1.1E-13	2.0E-11	TNFAIP6	-5.14	1.1E-10	1.1E-07	GPR1	3.76	4.2E-05	2.5E-03
NA	7.92	4.7E-13	6.5E-11	OLR1	-5.09	1.1E-04	3.0E-03	ECM2	3.74	1.6E-05	1.2E-03
IRF8	7.84	1.9E-14	4.2E-12	C3	-4.96	4.7E-13	1.9E-09	AMPD3	-3.73	4.5E-08	2.3E-05
PLA1A	7.82	4.0E-17	3.1E-14	MGAT3	-4.93	9.4E-06	5.1E-04	MME	3.72	6.7E-11	3.5E-07
MX1	7.81	9.2E-14	1.7E-11	WNT5A	-4.90	1.5E-11	2.0E-08	CXCL5	-3.72	3.6E-09	5.3E-06
SERPINB4	7.81	2.2E-10	1.2E-08	SP6	-4.85	3.4E-08	6.3E-06	NA	3.71	6.0E-05	3.1E-03
CD38	7.8	5.9E-12	5.5E-10	ACOD1	-4.81	9.5E-06	5.2E-04	LUCAT1	-3.71	1.1E-08	9.4E-06
SECTM1	7.77	1.3E-18	2.2E-15	CHI3L2	-4.80	2.2E-08	4.7E-06	CSPG5	-3.65	2.9E-08	1.8E-05
USP30-AS1	7.76	1.9E-12	2.2E-10	PI3	-4.79	9.1E-08	1.4E-05	HKDC1	3.63	8.2E-05	3.6E-03
IL12RB1	7.75	1.7E-11	1.4E-09	CCL8	-4.72	1.1E-12	3.2E-09	MYOCD	-3.62	3.2E-08	1.9E-05
EBI3	7.69	6.8E-11	4.3E-09	LINC00996	-4.69	2.1E-07	2.6E-05	LINC00996	-3.57	2.7E-06	3.7E-04
UBD	7.67	2.4E-11	1.9E-09	SELL	-4.66	1.8E-09	7.2E-07	INHBE	3.56	2.1E-04	6.7E-03
RSAD2	7.64	6.0E-12	5.5E-10	NA	-4.62	1.2E-10	1.1E-07	MGAT3	-3.48	1.8E-04	6.0E-03
CMPK2	7.64	2.2E-13	3.5E-11	STEAP2	-4.61	4.1E-10	2.5E-07	NIBAN1	3.48	1.3E-07	4.8E-05
TNFSF13B	7.62	1.6E-15	5.5E-13	DUOX2	-4.59	1.7E-07	2.2E-05	MGAT3	-3.48	1.8E-04	6.0E-03

1 Genes are classified as differential expressed when log2FC>1 and $p<0.05$.
Changes are ranked based on log2FC.

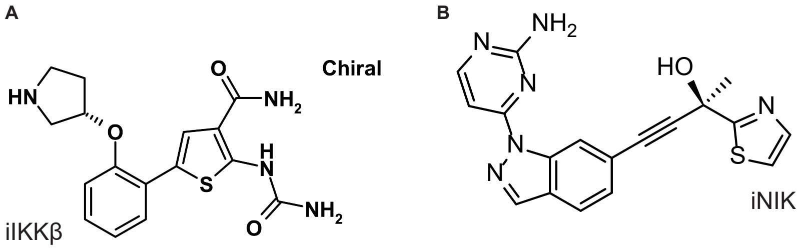

Supplementary Figure 1: Molecular structures of used inhibitors. (A) iIKKβ and (B) iNIK.

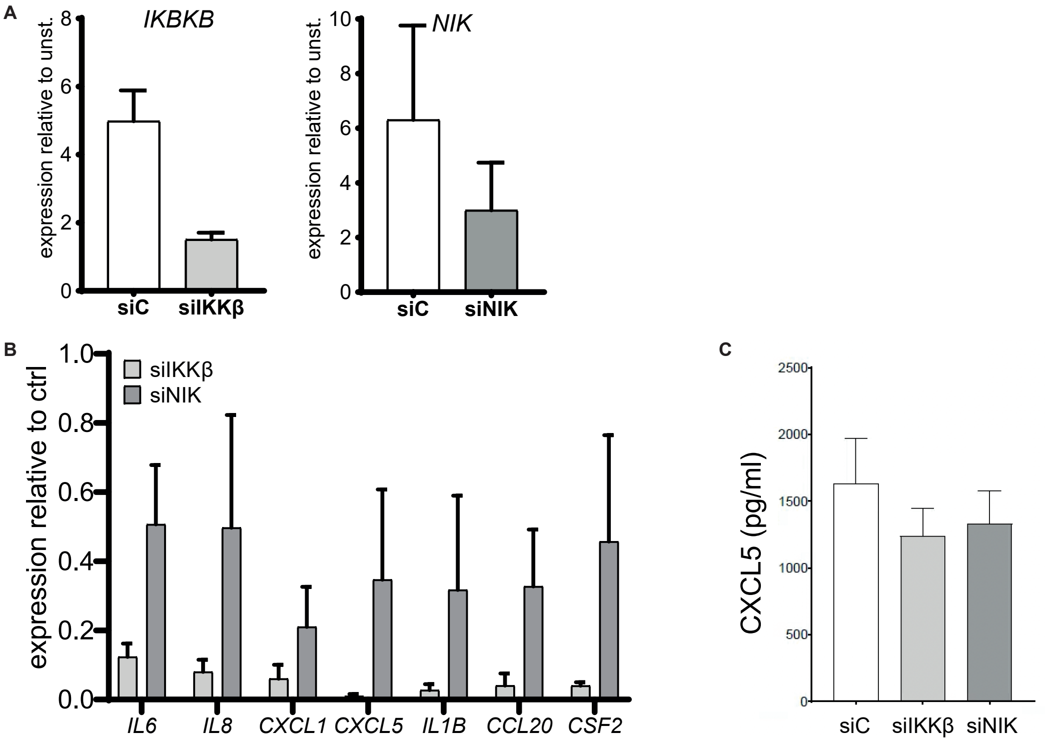

Supplementary Figure 2: Treatment with siRNA targeting IKKβ or NIK has similar effects as treatment with inhibitors. HUVECs were stimulated with T_m sup for 72h. (A) expression of IKBKB (in HUVEC treated with siIKKβ (left)) and NIK (in HUVECs treated with siNIK (right)) was analyzed to assess knock-down efficiency. Expression is shown relative to unstimulated (unst.) HUVECs (*N* = 3). (B) mRNA expression of indicated chemokines and cytokines in siRNA treated HUVECs. Expression is shown relative to siC treated HUVEC (*N* = 3). (C) ELISA was performed to measure CXCL5 production in siRNA treated HUVEC (*N*= 4).

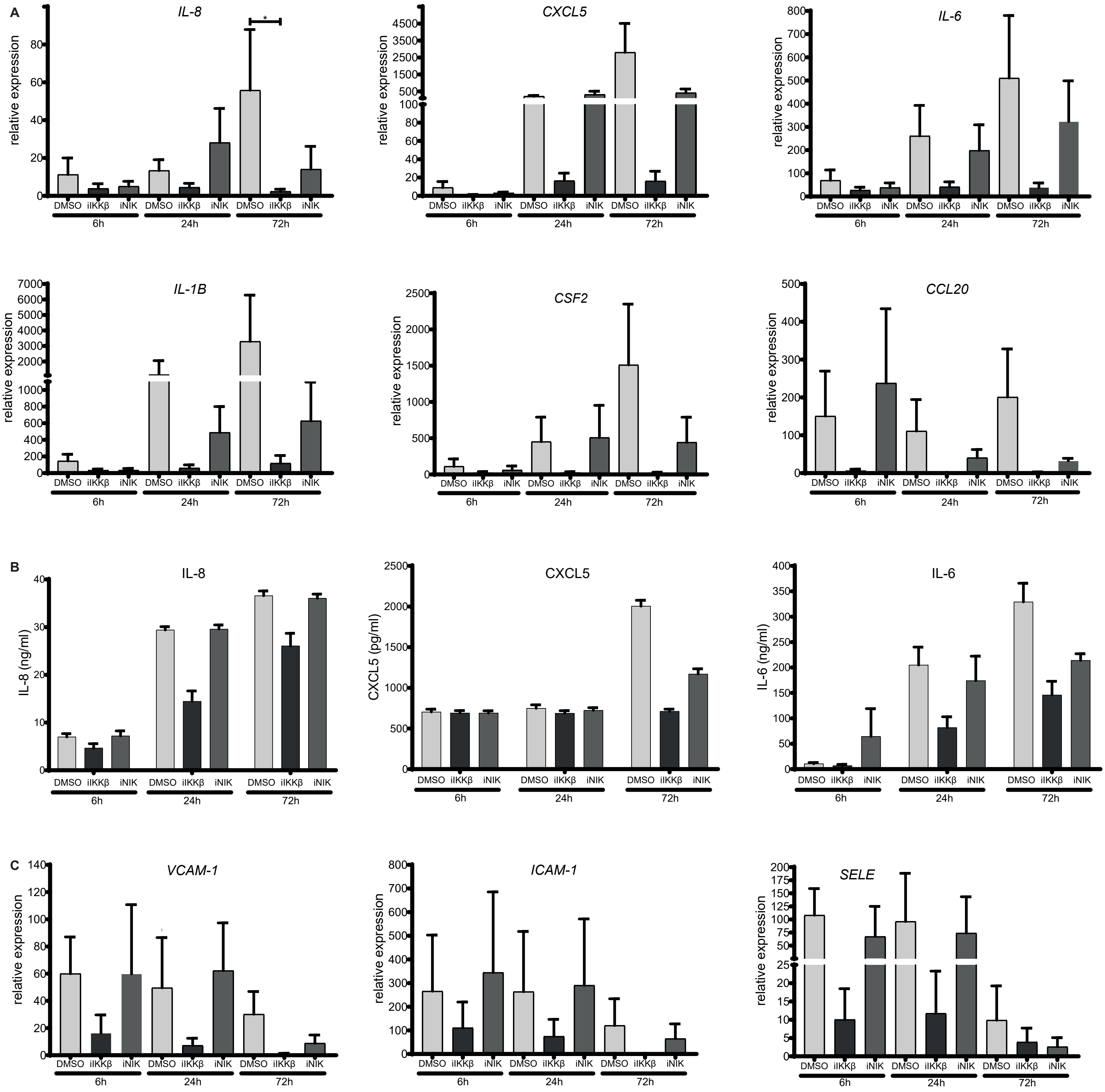

Supplementary Figure 3: Effects of IKKβ and NIK inhibitor on inflammatory mediators over time. HUVEC were treated with iIKKβ and iNIK and stimulated with T_m sup for indicated time points. (A) mRNA expression of indicated chemokines and cytokines. Expression is shown relative to unstimulated HUVECs (unst.) (*N* = 3). (B) ELISA was performed to measure protein production of indicated chemokines (*N*= 4). (C) mRNA expression of indicated adhesion molecules. Expression is shown relative to unstimulated HUVECs (unst.) (*N* = 3).
